# Survey on the implementation of the *Occupational Health and Safety Act* at an academic hospital in Johannesburg

**DOI:** 10.4102/curationis.v39i1.1524

**Published:** 2016-09-28

**Authors:** Muraga R. Foromo, Mary Chabeli, Mpho M. Satekge

**Affiliations:** 1 Department of Public Service and Administration, University of Johannesburg, South Africa

## Abstract

**Background:**

Despite the available research findings, recommendations and the *South African Occupational Health and Safety Act* (OHSA) (*Act* 85 of 1993), there are still challenges with regard to the implementation of selected sections and regulations of the OHSA. This is evidenced by the occupational injuries and illness claims registered with the compensation fund (South Africa, Department of Labour 1993).

**Objectives:**

To determine the extent to which the OHSA was implemented at an academic hospital in Johannesburg, from the senior professional nurses and nursing managers’ perspective, and to describe recommendations in order to facilitate the implementation of the Act.

**Methods:**

A contextual, quantitative, exploratory and descriptive survey was conducted. A purposive sampling method was used to select the participants that met the inclusion criteria. A structured Likert-scale questionnaire was used to collect data (Brink 2011). Stata version 12 was used to analyse the data. Cronbach’s alpha, with a cut-off point of 0.7 was used to test for internal consistency. Ethical considerations were strictly adhered to. Results are presented in the form of graphs, frequency distributions and tables.

**Results:**

The study revealed that overall there is 93.3% non-implementation of the selected sections and regulations of the OHSA. These results have serious implications on the health and safety of employees in the workplace.

**Conclusion:**

The study recommends that the replication of the study should be conducted in order to determine the extent of implementation of the selected sections and regulations of the OHSA in other government institutions.

## Introduction and rationale

According to the International Labour Organization (2003), occupational health and safety is defined as a discipline with a broad scope involving many specialised fields such as the promotion and maintenance of the highest degree of physical, mental and social well-being of workers in all occupations; the prevention amongst workers of adverse effects on health caused by their working conditions; the protection of workers in their employment from risks resulting from factors adverse to health; the placing and maintenance of workers in an occupational environment adapted to their physical and mental needs; and the adaptation of work. It is estimated that every year two million needlestick injuries occur in health-care workers worldwide (World Health Organization 2002). This is supported by Wicker *et al*. (2008), who indicated that 350 million people suffer from chronic HBV infection worldwide; 125 million people are infected with HCV and 33 million with HIV, making viral hepatitis and HIV two of the world’s greatest infectious disease in the workplace.

Studies have been conducted in different parts of the world on occupational health and safety in the workplace, especially on the subject of occupational injuries and diseases. In Europe, 60% of musculoskeletal problems were reported as the main work-related health problems, followed by 14% of stress, depression and anxiety (Van den Broek *et al*. 2011). In Brazil, some of the common occupational injuries and diseases are: pneumoconiosis, occupational cancer, occupational dermatitis, and work-related neurophysiological disorders such as stress, depression and burnout (Marziale & Hong 2005). In the Philippines, De Castro *et al*. (2009) conducted a study and found that amongst the health professionals, nurses were in the majority of those suffering occupational-related illnesses, with more than 78% experiencing back pain. In addition Tinubu *et al*. (2010) reported that work-related musculoskeletal disorders are common amongst health-care workers, with the nursing population that constitutes about 33% of the hospital workforce at particularly high risk, and accounting for 60% of nurses reporting work-related musculoskeletal disorders-related illnesses at the Ibadan hospital, in South-West Nigeria. A study regarding the needle stick injuries was conducted in Johannesburg and Soweto hospitals. The study revealed that (*n* = 102) 83% of the medical interns sustained percutaneous injuries. Of these 43% were from HIV-positive patients (Karstaedt & Pantanowitz 2001). The above empirical studies revealed that there is still a gap on the implementation of the *Occupational Health and Safety Act* (OHSA) in the public sector. An academic hospital in the Johannesburg is not unique as it is still encountering challenges on the implementation of the OHSA (*Act* 85 of 1993). This is evidenced by the report obtained from the hospital Injury on Duty register. The incidence rates in 2010 were 36%, in 2011, 49% and in 2012, 67% respectively. The report shows that there is an escalation of injuries every year, which is cause for concern. Furthermore, this report is supported by Nophale (2009), who conducted a study on reported needlestick injuries amongst health-care workers in regional hospitals in Free State Province, South Africa. The results of the study revealed that 90% of professional nurses sustained needlestick injuries, followed by 55.6% of doctors.

Based on the above studies it is evident that there is insufficient implementation of the OHSA (*Act* 85 of 1993) in the public sector.

## Problem statement

Regardless of the occupational health and safety legal frame work in place, the work environment including an academic hospital in Johannesburg still experience high incidence of occupational injuries due to insufficient implementation of the OHSA (*Act* 85 of 1993). This is evidenced by the above mentioned report obtained from the hospital Injury on Duty register. It is also supported by Kitt *et al*. (2006) who stated that health-care workers are at high risk of work-related illness and injury, facing a unique constellation of biological, chemical, physical, and ergonomic hazards, as well as psychological stress. The Department of Health has a draft occupational health and safety policy and a Guidelines booklet for health-care workers (2003). The OHSA (*Act* 85 of 1993) and the *South African Mine Health and Safety Act* (*Act* 29 of 1996), both require medical surveillance to be conducted and to manage the risk identified Tudor *et al*. (2014). According to Nophale (2009), employers are legally bound to provide a healthy and safe work environment to all employees and their surroundings. As part of the implementation of the OHSA employers are compelled to conduct medical surveillance once a year depending on the risks identified. However there is still a gap in the implementation of the OHSA (*Act* 85 of 1993) at an academic hospital in Johannesburg. The escalating incidence of injuries prompted the researcher to explore the implementation of the following selected sections and regulations of the OHSA (*Act* 85 of 1993), at an academic hospital in Johannesburg. Section 8: General duties of employers to employees; Section 14: General duties of employees at work; Section 17: Health and safety representatives; Section 19: Health and Safety Committee; Regulations on the reporting of incidents and occupational diseases; Regulations on recording and Investigation of incidents; Regulations on hazardous biological agents medical surveillance; and regulations on hazardous biological agents records-keeping.

## Objectives of the study

To determine the extent of implementation of the selected sections and regulations of OHSA (*Act* 85 of 1993) at an academic hospital in Johannesburg, from senior professional nurses and the nursing managers’ perspectives.

To describe recommendations to facilitate the implementation of the selected sections and regulations of the OHSA (*Act* 85 of 1993) at an academic hospital in Johannesburg.

## Literature review

The South African government has put more emphasis on the adoption of the primary health care approach in the delivery of health-care services in order to promote the availability of basic health-care services to all South Africans, both at the workplace and in the community (Zungu 2007). Section 24 (a) in the Bill of Rights states that, everyone has the right to an environment that is not harmful to their health or well-being, and Section 27(1) (a) further states that everyone has the right to health-care services (South Africa, Constitution of the Republic of South Africa 1996). The employee health and wellness programme, which includes the *Occupational Health Safety Act* (OHSA) (*Act* 85 of 1993), are measures specifically developed to promote these rights (South Africa, Department of Public Service and Administration, Employee Health and Wellness Strategic Framework 2008). South Africa, Department of Public Service and Administration, Public Service Regulation (2001) indicates that the head of department of government institutions shall establish and maintain a safe and healthy work environment for employees of the department (South Africa, Department of Public Service and Administration, Employee Health and Wellness Strategic Framework 2008).

Workers are at high risk for occupational injuries and illness, such as psychological stress, and biological, chemical, physical, and ergonomically related hazards (Kitt *et al*. 2006). Furthermore, Kielkowski, Rees and Bradshaw (2004) reported that in South Africa, musculoskeletal disorders and respiratory diseases are the most common causes for staying away from work. The implementation of the OHSA aids in ensuring and enhancing better health for all employees in the workplace, thus promoting a high morale and increased productivity.

The International Labour Organization recommends that occupational health services should protect the health of employees against potential hazards at work, and also maintain or improve health by education and promotion of primary health care as well as mitigation of associated risks (Tudor *et al*. 2014). A number of regulations in the OHSA are under continuous review to promote safer working conditions in several sectors. In addition, the *Compensation of Occupational Injuries and Diseases Act* (*Act* 130 of 1993) is applicable in order to ensure the compensation of employees who sustained occupational injuries and illness or contracted occupational diseases associated with their work (South Africa, Department of Labour 1993).This is in consistent with the implementation of the OHSA in the workplace.

Studies have been conducted in different parts of the world on occupational health and safety in the workplace, especially on the subject of occupational injuries and diseases. In Europe, 60% of musculoskeletal problems were reported as the main work-related health problems, followed by 14% of stress, depression and anxiety (Van den Broek *et al*. 2011). The report was further supported by Silva and Steinbuka (2010) on a European Working Conditions Survey in which backache, muscular pain, and stress were often reported by workers. In Brazil, some of the common occupational injuries and diseases are: pneumoconiosis, occupational cancer and occupational dermatitis, and work-related neurophysiological disorders such as stress, depression and burnout (Marziale & Hong 2005). According to Tinubu *et al*. (2010), work-related musculoskeletal disorders are common amongst health-care workers, with the nursing population that constitutes about 33% of the hospital workforce at particularly high risk, and accounting for 60% of nurses reporting work-related musculoskeletal disorders-related illnesses in the Ibadan hospital, in South-West Nigeria. De Castro *et al*. (2009) conducted a study in the Philippines and found that amongst the health professionals, nurses were in the majority of those suffering occupational-related illnesses, with more than 78% experiencing back pain.

South Africa is one of the countries that can afford better occupational health services than its African neighbours as is well better resourced (Mazars *et al*. 2013). The reengineering of the health-care system also put South Africa in a better position as compare to its neighbours. In the 2011 and 2012 financial year, the compensation fund paid the claims in the amount of R 2431, 578, 235 for work-related accidents reported (South Africa, Department of Labour 2012). This is in line with the implementation of the OHSA to compensate the deserved injured employees as stipulated in the *Act*. The highest incidence of occupational injuries and fatalities are found in the mining, construction, and iron and steel sectors as well as the agriculture sectors, the most commonly reported occupational diseases were noise-induced hearing loss, pneumoconiosis, asthma and skin diseases (South Africa, Department of Labour 2012). The Department of Health has a draft occupational health and safety policy and a Guidelines booklet for health-care workers (2003). The OHSA (*Act* 85 of 1993) and the *Mine Health and Safety Act* (*Act* 29 of 1996) both require medical surveillance to be conducted and to manage the risk identified (Tudor *et al*. 2014). According to Nophale (2009), employers are legally bound to provide a healthy and safe work environment to all employees and their surroundings. As part of the implementation of the OHSA, employers are compelled to conduct medical surveillance once a year depending on the risks identified.

Based on the above studies it is evident that there is insufficient implementation of the OHSA (*Act* 85 of 1993) in the public sector. Hence, this study seeks to explore the *Act’s* implementation of the following selected sections and regulations of the OHSA. Section 8: General duties of employers to their employees; Section 14: General duties of employers at work; Section 17: Health and safety representatives; Section 19: Appointment Health and Safety Committee. Adherence to regulations on the reporting of incidents and occupational diseases; regulations on the recording and investigation of incidents; regulations on hazardous biological agents’ medical surveillance, and regulations on hazardous biological agents’ records-keeping.

## Research design and method

### Design

A contextual, quantitative, exploratory and descriptive design was used in order to survey the implementation of the OHSA and to describe the recommendations to facilitate the implementation of the selected sections and regulations of the Act at an academic hospital in Johannesburg.

### The setting

The setting for this study took place in the wards of the academic hospital. The hospital is situated in a central suburb of Johannesburg, with bed occupancy of 1 088. The hospital was easily accessible by the researcher, as the researcher was one of the employees at the time of conducting the research. The selected nursing wards were chosen based on the highest number of occupational incidents reported.

### Population

Somekh and Lewin (2006) refer to a research population as all the people and phenomena that are relevant to the study, from which a circumscribed sample will be selected for research. Furthermore, Burns and Grové (2009) refer to all elements that meet the sample criteria for inclusion in a study. In this study, population consists of 178 senior professional nurses, 70 nurse managers, and 37 wards in the academic hospital under study.

### Sample and sampling method

Burns and Grové (2009) indicate that sampling involves selecting a group of people with which to conduct a study, whilst Houser (2012) defines sampling as the potential participants who meet the description of the population accessible to the researcher. In this study, a purposive sampling method was used to select nursing wards and specialised units, and senior professional nurses and nursing managers who were on duty during the time of data collection, and also had 2 years of working experience in the same wards. A total number of 37 wards (20 specialised units and 17 general wards) were selected to participate in the study based on a high number of incidents reported; hence a purposively sampling method was used. Seventy-five senior professional nurses and 52 nurse managers who were on duty took part in the research survey. Two participants did not meet the criteria. Participation in this study was based on the willingness of participants to take part in the study. Participants were informed about the purpose and method of the research.

### Data collection instrument

The data collection tool was developed by the researcher derived from the OHSA (*Act* 85 of 1993). The questionnaire consisted of 2 sections. Section A dealt with the demographic data, whilst Section B dealt with 4 sections and 4 regulations which consisted of 45 items derived from the OHSA, dealing with the survey on the extent of the implementation of the selected sections of the OHSA by the senior professional nurses and nursing managers at an academic hospital in Johannesburg. Section B was further divided into 4 sections and 4 regulations as follows: (1) Section 8: General duties of employers to their employees consisted of 21 items; (2) Section 14: General duties of employers at work consisted of 3 items; (3) Section 17: Health and safety representatives consisted of 4 items; (4) Section 19: Appointment of Health and Safety Committee consisted of 4 items; (5) Adherence to regulations on reporting of incidents and occupational diseases consisted of 2 items; (6) Regulations on recording and investigation of incidents consisted of 4 items; (7) Regulations on hazardous biological agents’ medical surveillance consisted of 3 items; and (8) regulations on hazardous biological agents’ records-keeping consisted of 3 items. The selected sections and regulations items of the OHSA in the questionnaire were verified by the senior statistician from higher institution for data analysis.

### Data collection process

A structured, Likert-scale questionnaire was used to collect data (Brink 2011) from senior professional nurses and nursing managers concerning implementation of the selected sections and regulations of the OHSA (*Act* 85 of 1993). The data collection Likert-scale ranged from 1 to 5, indicating never, rarely, sometimes, frequently, and all the time respectively. Forty-five items of the selected sections and regulations from the OHSA (*Act* 85 of 1993) were used. Data was collected by the researcher over a period of two months. Senior professional nurses and nursing managers who were on duty at the time of the survey took part in the research. The survey was conducted during afternoons, when ward activities were reduced. Self-administered hardcopy questionnaires were used for the data collections. A total number of 140 questionnaires were distributed and the respond rate was 129 (92%).

### Validity and reliability of the study

Validity refers to whether the instrument actually measures the accuracy of the concept in question (De Vos *et al*. 2002). The authors’ further state that validity measures the degree to which the instrument is doing what it is intended to do to ensure validity of a study. Content validity for this study was ensured by using the questionnaire derived from the existing selected sections and regulations of the OHSA.

Reliability is a measure that denotes the consistency of a measure obtained by the use of a particular instrument, and is an indication of the extent of random error in the measurement method (Burns & Grové 2009). The items of the questionnaire used are reliably testing the envisaged latent construct, since the Cronbach`s alpha is 0.9583 (Table 1). In this study, the researcher used Cronbach`s alpha and pilot study to ensure reliability. Ten participants took part in the pilot study. The result of the pilot study was not included in the study. All the participants confirmed that the questions were well understood and thus the feasibility of the instrument was established.

**TABLE 1:** Professional category on gender.

Professional category	Gender				Total	
	Male		Female			
	*N*	%	*N*	%	*N*	%
Senior professional nurses	12	16	63	84	75	100
Nursing managers	5	9.61	47	90.38	52	100
**Total**	**17**	**13.4**	**110**	**86.6**	**127**	**100**

## Data analysis

Epi-Info V6 was used for data encoding. Data was transferred from Epi-format to Stata-format, wherein analysis was performed through Stata version 12. The analysis was performed at 95% confidence limit. The Pearson chi-square test was used to test for association between any two categorical variables. Frequency tables were generated to assess the distribution of all categorical variables. Cronbach’s alpha, with a cut-off point of 0.7 was used to test for internal consistency. All results are presented in tabular format and graphs. The total number of participants was 129, of which 2 were excluded, as they did not meet the criteria.

## Ethical considerations

The researcher used the ethical standards for nurses to ensure ethical considerations, which are based on human rights that need to be protected in research, such as the right to self-determination, privacy, anonymity and confidentiality, to fair treatment and to being protected from harm or discomfort (Brink 2011). Approval was obtained from the Higher Degree Committee and the Ethics Committee of a higher institution, prior to conducting the study. Permission to conduct the study was also obtained from the chief executive officer of the urban academic hospital. In this study, the researcher at all times adhered to ethical principles that respect and protect human rights. The right of participants to withdraw from the research at any time was also ensured.

## Results and discussion

### Biographical information

Table 1 indicates that all participants (*n* = 127) disclosed their gender, of which 13.4% (*n* = 17) were males, whilst the majority 86.6% (*n* = 110) were females. This was not a surprise, as traditionally the nursing profession is considered to be mainly a female profession (Bruce, Klopper & Mellish 2011). However, gender representation does not have any impact on the implementation of the OHSA in a hospital setting. Hardcopy questionnaires were used for data collection. A total of 140 questionnaires were distributed. The response rate of the participants was 92% (*n* = 129). Of the 127 participants, 126 participants disclosed their qualifications, 74 were senior professional nurses and 52 were nursing managers. Furthermore, 121 participants disclosed their ages whilst 6 participants did not.

## Discussion of the findings

Selected sections of the OHSA (*Act* 85 of 1993)

### Section 8: Duties of employers to their employees

#### Sub-theme 1.1: Provision of emergency escape exists with clearly visible signage

The study revealed that 49% (*n* = 62) of the participants indicated that emergency exit escape routes were not or rarely free from obstacles. According to the OHSA, the escape routes leading to emergency exits should always be free of any obstacles. Seleka and Oladele (2013) indicated that every employer should ensure that, where appropriate, all emergency exit routes should be free from obstruction. These will aid in case of emergency to minimise the stampede towards the exit door. Furthermore, this is supported by Koesterich (2011), who conducted a study on reviewing, assessment and prioritisation of an occupational health and safety management system in a veterinary teaching hospital in the US. The study found that the hospital was considered the best in the State of Colorado in terms of compliance and implementing the OHSA. The hospital follows the OHSA standards as required, such as emergency escape procedures, with emergency escape routes clearly visible and free from obstruction.

It is recommended that the escape routes leading to emergency exits should be free of any obstacles, as required by the OHSA. This is further supported by Hu *et al*. (1998), who conducted a study on employers’ awareness and compliance with occupational health and safety regulations in Taiwan, where a majority of employers (79%) indicated that emergency exit routes were free of obstruction and that there were posted safety warnings in their workplaces.

#### Sub-theme 1.2: Maintenance of housekeeping cleaning methods of storage rooms

The study revealed that 73% (*n* = 90) of the participants indicated that the housekeeping methods of storage rooms were never or rarely maintained or implemented. This is non-compliance with the OHSA by both senior professional nurses and nursing managers. According to the OHSA, housekeeping methods mean that every item should be in its original place. Furthermore, Alli states: ‘[*T*]he employer shall make available and maintain an unimpeded workplace for every employee; keep every indoor workplace clean, orderly and free of materials, tools and similar things which are not necessary for the work done in such workplaces’ (Alli 2008:66).

Worksafe Victoria (2007) explained that:

a safe and healthy workplace and compliance with the law does not happen by chance or guesswork; good health and safety is all about eliminating and controlling hazards and risks. A safe and healthy environment is best achieved by a proper consideration of the sources of harm and prevents the harm from occurring. (p. 57)

#### Sub-theme 1.3: Hazards procedures followed on clean-up of bloods and any other spilled fluids or slipping hazards

A high percentage (73%; *n* = 90) of the participants indicated that the clean-up of blood and any other spilled fluids and slipping hazards procedures were never or rarely followed. Based on the above results, it is evident that the health and safety of patients and health-care workers at an academic hospital under study are at risk. Wicker *et al*. (2008) state that health-care workers make up only 0.6% of the global population and that the contribution to hepatitis B, hepatitis C and HIV and AIDS infections on a global level is on the increase. However, health-care workers are at high risk of preventable infections from blood-borne pathogens, owing to their occupational exposure to infected blood and body fluids (Wicker *et al*. 2008). Nophale (2009) indicated that health-care workers are exposed to infectious blood and other body fluids in the course of their work. Consequently, they are at risk of occupational exposure and the transmission of viruses, including the hepatitis B virus, hepatitis C virus, and HIV. Since the infection of HIV and hepatitis is associated with body fluids it is vital for health-care workers to practice procedures on the clean-up of blood and any other spilled fluids when in contact with body fluids to prevent further infections amongst health-care workers

In addition, it is recommended that there is a need to provide nursing personnel working in the wards at an academic hospital with a written Standard Operating Procedure (SOP) on the clean-up of blood or any other spilled fluids in the wards. This is further supported by Alli (2008), who states that employers should ensure that adequate procedures are in place for the routine care, cleaning and disinfection of environmental surfaces, and other frequently used or potentially contaminated surfaces.

#### Sub-theme 1.4: Following of waste disposal methods in the wards

The study revealed that a majority (74%; *n* = 94) of the participants indicated that waste disposal methods were never or rarely followed, whilst 77% (*n* = 98;) of the participants responded that appropriate packaging for hazardous medical waste were never provided and 76% (*n* = 97) of the participants indicated that they were never or rarely trained on universal precautionary measures, for example the closing and removal of sharps containers. Furthermore, 77% (*n* = 98) of the participants indicated that waste was not or rarely separated correctly, with clearly labelled packaging. According to Boskey (2010):

universal precautions refer to certain infection-control steps that medical professionals take to reduce the risk of transmitting HIV and other infectious diseases. The scientific basis of universal precautions is that individuals should treat any blood or bodily fluid as though it contains HIV, hepatitis, or other infectious agents.

The author further explained that universal precautions assume that all bodily fluids are dangerous and health professionals should treat them with caution. The OHSA mandated the use of universal precautions as a form of infection control in the early 1990s, after it became clear that HIV spread through exposure to blood and certain other bodily fluids (Boskey 2010).

In addition, all hazardous waste must be properly labelled; the labels must have an instruction on the back and always be attached to the primary waste containers (Stanford University 1998). The OHSA (*Act* 85 of 1993) states that all hazardous waste should be properly contained and controlled to prevent the spread of contamination in the workplace (South Africa, Department of Labour 1993).

The training of the workers themselves in occupational health and safety issues, as well as their rights within the workplace has been an important part of improving working conditions. When workers are aware of hazards and understand the code of conduct, they can initiate discussions with business owners that result in positive change within the work environment (Carothers, Foad & Denomy 2009). Furthermore, Alli (2008) argues that employers should ensure that all waste that can cause harm by its exposure is disposed of at a marked, designated area, and in a manner that does not cause hazard in the workplace. McDiarmid (2014) stated that biological hazards are encountered in all health-care settings, and include airborne and blood-borne pathogens such as the agents that cause tuberculosis, severe acute respiratory syndrome, hepatitis and HIV infection. Therefore, it is recommended that hazardous waste should be separated from non-hazardous waste, and clearly labelled or colour-coded. Waste management SOPs should be developed and made available to all employees dealing with waste. Nursing personnel need to be trained on the universal precautions method during their induction, and thereafter on a regular basis. This is supported by Alli (2008), who recommended that a manual for the appropriate decontamination and disinfection procedures should be developed, and that continuous training on universal precautions should be an ongoing process.

#### Sub-theme 1.5: Measures practised for promotion of good hygiene in the ward

A majority of the participants (78%; *n* = 100), indicated that the promotion of good hygiene in the nursing ward was never or rarely practised. Work-related illness and infections usually occur due to the nature of the work nurses do (Katsuro *et al*. 2010). Therefore it is recommended that the promotion of good hygiene in nursing wards should take place at all times. One of the broad objectives of occupational health programmes in the workplace is to promote, support, and provide a healthy and productive, highly functioning workforce to the employer (Nophale 2009).

#### Sub-theme 1.6: Provision of personal protective equipment for nurses by nursing managers

More than half of the participants (63%; *n* = 80) indicated that nurses were not or rarely provided with personal protective equipment (PPE) by the employer. Katsuro *et al*. (2010) explained that accidents do not arise from a single cause but from a combination of factors which act simultaneously and are often caused by unsafe conditions. A similar study conducted by Bakibinga (2012) in Uganda found that nurses working in the public sector often have to work without basic protective gear, such as gloves, due to stock-outs. Lack of protective clothing and equipment exposes workers to occupational hazards and diseases, thereby reducing their efficiency and productivity (Katsuro *et al*. 2010). Furthermore, the OHSA compels the employer to provide PPE to employees who are exposed to hazards during their work activities (South Africa, Department of Labour 1993). It is recommended that employers should provide the correct PPE to employees who are at risk whilst performing their duties, as required by the OHSA. This is further supported by Katsuro *et al*. (2010), who stated that the lack of PPE reduces employee morale, which results in low productivity because workers become less willing to work; therefore employers should consider the health of their workers before productivity by providing the necessary PPE to their workers.

#### Sub-theme 1.7: Availability of written occupational health and safety policy in the ward

The study revealed that more than half of participants 76% (*n* = 97) indicated that there was no or rarely written policy on health and safety in the ward and 90% (*n* = 114) of the participants revealed that a policy on occupational health and safety was not or rarely displayed in the nursing ward. The non-availability of the occupational health and safety policy in the nursing wards is an indication that the senior professional nurses and nursing managers do not comply with the implementation of the occupational health and safety measures in their wards. Legal obligations compel employers in some jurisdictions to implement practices that ensure workplace equity, health and safety. Hermanus (1999) defined policy as the norms for achieving safe and healthful workplaces, which typically include a commitment to the prevention of occupational injury. Furthermore, Joan (2011) explained that the laws, regulations, and standards adopted or endorsed in many countries prohibit various forms of workplace discrimination and require health and safety goals to be achieved in ways that do not infringe workers’ other rights and interests. In addition, it is recommended that the management at an academic hospital should develop an occupational health and safety policy which will address health and safety matters in the workplace. This policy should be signed by the chief executive officer and be displayed on all nursing wards’ notice boards, as well as at the entrance of the hospital building, at the canteen and the auditorium, and be visible to everybody entering the hospital. Moreover, the OHSA (*Act* 85 of 1993) states that the employer shall develop in writing an occupational health and safety policy concerning the protection of the health and safety of employees at work, including the reviewing of that policy (South Africa, Department of Labour 1993).

#### Sub-theme 1.8: Ensuring of a working environment that is safe and without risk by the employees

The study showed that most of the participants 976%; *n* = 96) indicated that their employer rarely ensured a safe working environment without risk, whilst 82% (*n* = 104) of the participants responded that their employer never raised awareness of measures that need to be taken to ensure compliance with the OHSA in the hospital environment. Hattingh and Acutt (2003) further explain that it is the employer`s general duty to provide and maintain, as far as is reasonably practicable, a working environment that is safe and without risk to health. It is therefore recommended that the employer should provide a safe and healthy working environment and raise an awareness campaign on measures to be taken to ensure compliance with the OHSA, to all employees of an academic hospital. This is further supported by Katsuro *et al*. (2010), who indicate that occupational health and safety statutory instruments state that it is the employer’s obligation to provide a safe working environment for the workers. In addition, Seleka and Oladele (2013) state that every employer should conduct his undertaking in such a manner as to ensure that persons other than those in his employment who may be directly affected by their activities are not thereby exposed to health hazards.

#### Sub-theme 1.9: Provision of an opportunity for training on the OHSA by the employer

A majority of participants (61%; *n* = 77;) indicated that their employer rarely or never provided an opportunity for OHSA training, and another 61% (*n* = 77;) participants responded that their employer rarely provided supervision on the implementation of the OHSA in the ward. The nurses’ ability to provide safe, healthy and high quality care can be dependent upon their abilities to reason, think, and judge, which can be limited by lack of experience and knowledge (Benner, Hughes & Stuphen 2008). It is therefore recommended that senior professional nurses and nursing managers should be given the opportunity of OHSA (*Act* 85 of 1993) training, supervision and implementation in the ward. In order to encourage the implementation of the OHSA at an academic hospital, nurses should be given the opportunity to be capacitated on matters relating to their health and safety. This will assist them to make sound judgements in the course of their daily activities. This is supported by Alli (2008), who stated that:

training in occupational health and safety should not be treated in isolation; it should feature as an integral part of job training and be incorporated into daily work procedures on the shop floor.

### Section 14: General duties of employees

A majority (75%; *n* = 95) of the participants indicated that employees rarely or never take care of their own health and safety or that of others, and do not carry out lawful orders in the interest of occupational health and safety. Similarly, 75% (*n* = 95) of the participants responded that employees never or rarely obey health and safety rules and procedures. This practise behaviour raises a serious of concern as the acts compel every employee to report any unsafe work environment before the end of the shift as part of taking responsibility for own health and of others. According to Bennet (2002):

every individual in life, whether employed or [*unemployed*], both [*in*] the workplace and outside the workplace, has the need to be safe. Employees, as mature individuals, are responsible for every decision they make with regard to securing their own health and safety in every social setting.

It is therefore recommended that all employees be trained on their general duties as stipulated in the OHSA. This is further supported by Machabe and Indermun (2013), who explain that Section 14 of the OHSA requires that every employee at work should take reasonable care and safety of themselves and of others who may be affected by their acts or omissions.

### Section 17: Health and safety representatives

In this section the majority of participants (75%; *n* = 94) indicated that health and safety representatives were appointed with a signed letter by the CEO of the hospital, whilst 52% (*n* = 66) of the participants indicated that health and safety (HS) representatives were never or rarely trained regarding their roles. Furthermore, 64% (*n* = 81) of the participants showed that the HS representatives did not or rarely carry out their delegated tasks as required by the OHSA, whilst 66% (*n* = 84) of the participants revealed that the HS representatives never or rarely attend the HS Committee meetings. The appointment of health and safety representatives without explaining and training their roles is malicious compliance, as it will be difficult for the HS representatives to execute their responsibilities as stipulated in the OHSA. According to Hattingh and Acutt (2003), the employer has a duty to appoint in writing the occupational HS representatives and explain to all employees their responsibilities within the organisation after being elected by their co-workers. It is therefore recommended that all HS representatives should be trained or workshop on their responsibilities as stipulated in the OHSA.

### Section 19: Appointment of the Health and Safety Committee

In this section 82% (*n* = 103) of the participants indicated that HS Committee members were appointed with a letter signed by the CEO of the hospital, whilst 51% (*n* = 64) of the participants indicated that the HS Committee did not hold meetings. Furthermore, 61% (*n* = 77) of the participants showed that the HS Committee members never or rarely function as required by the OHSA, whilst 65% (*n* = 81) of the participants revealed that the HS Committee members never or rarely identify and address shortfalls in order to prevent their recurrence. An accredited service provider for training on OHSA should be in house to train all appointed HS representatives and committee members regarding their roles and responsibilities in order for them to be functioning optimally. Hattingh and Acutt (2003) further explain that the HS Committee members should ensure that safety standards are set and followed in the workplace. It is therefore recommended that HS Committee members should be trained or workshop on their responsibilities as stipulated in the OHSA (*Act* 85 of 1993).

### Adherence to the regulations on reporting of incidents and occupational diseases

A majority (76%; *n* = 96) of the participants indicated that the reporting of any incident, near-miss or unsafe situation to the employer or a HS representative is never or rarely implemented, whilst 94% (*n* = 119) of the participants revealed that informing the inspector from the Department of Labour about incidents that require reporting was not or rarely done. It is the responsibilities of an employer to report any occupational injuries within 7 days and occupational diseases within 14 days to the Compensation Commissioner for further management as required by the OHSA. An employee is entitled to the benefits of the Compensation for *Occupational Injuries and Diseases Act* (*Act* 130 of 1993) if an employee sustains an injury as a result of an incident which arose from related duties and in the course of duties, or if said employee contracts an occupational disease for which compensation is payable as a result of the nature of work activities (Nophale 2009). The employer is compelled to report any incidents and occupational diseases within the stipulated time frames as required by the OHSA; if the employer does not adhere to the acts, they will be fined or may even face imprisonment. It is therefore recommended that employees be encouraged to report any incidents or near-misses to their representatives or supervisors before the end of the shift as required by the OHSA and that the employer should report any reportable injuries and occupational diseases to the Compensation Commissioner within the stipulated time to avoid fined or imprisonment.

### Adherence to the regulations on the recording and investigating of incidents

A majority of participants (77%; *n* = 98) indicated that incidents that had occurred in the area of their responsibility were not or rarely reported, whilst 88% (*n* = 113) of the participants revealed that all incidents that occurred were never or rarely fully investigated and recorded, and 93% (*n* = 118) of the participants indicated that all identified causes of incidents were never or rarely eliminated. Furthermore, most of the participants (90%; *n* = 114) revealed that the HS Committee never investigated incidents that occurred in the workplace. Not investigating and recording incidents that had occurred in the workplace is a non-compliance on the implementation of the OHSA which can result in recurrence and cause damage to the employees and the organisation. Therefore, it is imperative that every incident be investigated and recorded to prevent a recurrence and eliminate the risks. According to Katsuro *et al*. (2010), if incidents are not reported or recorded, they will not be known to the management, which results in accidents going unnoticed; thus as a result no measures are taken to prevent a recurrence of the same accidents in the future. The purpose of the investigation of incidents is not to find fault, but rather to find facts in order to prevent further occurrences (Gezairy 2001). It is therefore recommended that employees should report any incident at the workplace to their supervisors or occupational HS representatives and that the employer should investigate as required by the OHSA and eliminates the risks. This is supported by Osborn and Williams (2004), who state that the report, records, and the investigation of the incidents assist the employer to put in place mitigation factors in order to prevent recurrence.

### Adherence to the regulations on hazardous biological agents: Medical surveillance

More than half of participants (77%; *n* = 98) indicated that employees with occupational-related diseases were not or rarely managed, whilst 78% (*n* = 99) revealed that pre-employment medical examinations were never or rarely conducted on nurses before the commencement of their employment; most of the participants (78%; *n* = 99) also revealed that physical examinations and any other essential examinations were never or rarely conducted as stipulated by OHSA or as required by the occupational health practitioner. Occupational medical examinations are medical examinations of employees that are aimed at the prevention of health risks that may arise from their occupation (Nolting *et al*. 2007). It is recommended that employees are given pre-medical examinations and any other essential examinations by the occupational health practitioner prior to their commencement of employment. This is supported by Alli (2008), who states that employee health surveillance can detect and identify any abnormalities in the employee’s health that could relate to their work environment.

### Adherence to the regulations on hazardous biological agents: Records-keeping

A majority of participants (79%; *n* = 100) indicated that medical surveillance records are never or rarely kept safely for 40 years, whilst 79% (*n* = 100) of the participants indicated that medical records were not or rarely accessed by the medical practitioner, and most participants 95% (*n* = 120) revealed that it was not possible to access the medical records of an employee without his or her consent. The employer should keep the records of all medical surveillance assessment reports; and personal medical records should be made available only to an occupational health practitioner or, if not an occupational health practitioner, then a formal written consent from the employee should be needed to allow any perusal of his or her records (Hattingh & Acutt 2003). Keeping records for 40 years will aid the hospital in producing them in times of litigations and verify whether the injuries and diseases were acquired in the workplace when the employees are no longer working or have retired. Failure to produce these documents will put the hospital management in a negative light before the law without a proof. It is recommended that the employer should keep all medical surveillance records for a period of 40 years and comply with the employees’ medical records confidentiality, as required by the OHSA (*Act* 85 of 1993) to prevent unnecessary litigations.

## Limitations of the study

Data were collected only from the senior professional nurses and nursing managers who were on duty at an academic hospital in Johannesburg.

## Recommendations

Recommendations are made in terms of nursing education, nursing practice and nursing research. Over and above the specific recommendations emanating from the study, the researcher recommends that short courses should be developed based on the described recommendations of the study. Such courses will probably better inform nurses and improve their skills in being innovative in the implementation of the OHSA for compliance in all nursing wards.

A well-structured occupational HS programme including awareness and strong infections control programmes should be established in the hospital according to the described recommendations, and be offered to newly recruited nurses during their orientation programme. A feasible, practical human resource development programme should be reinforced to improve the continuing education of practising nurses through in-service training and workshops. The effectiveness of the educational programme should be monitored. Since the study is contextual in nature it is recommended that replication of the study should be conducted in order to determine the extent of implementation of the selected sections and regulations of the OHSA in other government institutions. A qualitative study is further recommended to reveal and to obtain qualitative data of participants regarding their experiences of the implementation of OHSA. The Gauteng Department of Health should secure bursaries for post-graduate nurses in order to encourage ongoing research regarding the implementation of the OHSA (*Act* 85 of 1993).

## Conclusion

The purpose of this study was to determine the extent of implementation of the OHSA at an academic hospital in Johannesburg from the senior professional nurses and nursing managers’ perspective, and to make recommendations to facilitate the implementation of the *Act*. The study revealed that from the selected sections and regulations of the OHSA, 95.4% of section 8; 100% of section 14; 75% of both sections 17 and 19 were not implemented, whilst 100% of all 4 regulations were also not implemented. Furthermore, the study revealed that overall there is 93.3% non-implementation of the selected sections and regulations of the OHSA, where 42 of the 45 items were not implemented. These results have serious implications on the HS of employees in the workplace. The recommendations made in the study will assist to facilitate the implementation of the selected sections and regulations of the OHSA (*Act* 85 of 1993) at an academic hospital in the Johannesburg.
